# Relationship between the structure and function of the transcriptional regulator E2A

**DOI:** 10.1186/s40709-021-00146-5

**Published:** 2021-07-16

**Authors:** Jia-Jie Liang, Hu Peng, Jiao-Jiao Wang, Xiao-Hui Liu, Lan Ma, Yi-Ran Ni, Huai-Jie Yang, Yan-Qiong Zhang, Wen-Bing Ai, Jiang-Feng Wu

**Affiliations:** 1 Medical College, China Three Gorges University, 8 Daxue Road, Xiling District, Yichang, 443002 China; 2 Institute of Organ Fibrosis and Targeted Drug Delivery, China Three Gorges University, 8 Daxue Road, Xiling District, Yichang, 443002 China; 3 Hubei Key Laboratory of Tumor Microenvironment and Immunotherapy, China Three Gorges University, 8 Daxue Road, Xiling District, Yichang, 443002 China; 4 The People’s Hospital of China Three Gorges University, 31 Huti Subdistrict, Xi Ling District, Yichang, 443000 Hubei China; 5 The Yiling Hospital of Yichang, 32 Donghu Road, Yi Ling District, Yichang, 443100 Hubei China

**Keywords:** E2A, bHLH domain, Activation domain, Structural basis

## Abstract

E proteins are transcriptional regulators that regulate many developmental processes in animals and lymphocytosis and leukemia in *Homo sapiens*. In particular, E2A, a member of the E protein family, plays a major role in the transcriptional regulatory network that promotes the differentiation and development of B and T lymphocytes. E2A-mediated transcriptional regulation usually requires the formation of E2A dimers, which then bind to coregulators. In this review, we summarize the mechanisms by which E2A participates in transcriptional regulation from a structural perspective. More specifically, the C-terminal helix-loop-helix (HLH) region of the basic HLH (bHLH) domain first dimerizes, and then the activation domains of E2A bind to different coactivators or corepressors in different cell contexts, resulting in histone acetylation or deacetylation, respectively. Then, the N-terminal basic region (b) of the bHLH domain binds to or dissociates from a specific DNA motif (E-box sequence). Last, trans-activation or trans-repression occurs. We also summarize the properties of these E2A domains and their interactions with the domains of other proteins. The feasibility of developing drugs based on these domains is discussed.

## Background

The E protein family, also known as the class I basic helix-loop-helix (bHLH) family, is the transcription factor family that contains the bHLH domain [1]. The family includes six members, with E12, E47, E2-2, and HEB expressed in mammals, daughterless expressed in *Drosophila melanogaster*, and HLH-2 expressed in *Caenorhabditis elegans* [2]. These transcription factors have wide-ranging roles in the immune system and regulate the recombination of immunoglobulins, the differentiation of most lymphocytes, the function of lymphoid tissues, and the development of most lymphoid tissues. E proteins generally regulate these immune system functions by functioning as transcriptional regulators and participating in a very large transcriptional network that affects cellular growth, differentiation and proliferation [3].

E12 and E47, collectively known as E2A, were the first E proteins identified [3]. E2A was initially identified as a transcription factor that binds to the κE2/μE5 site located in the human immunoglobulin kappa chain enhancer. The E2A gene encodes two E2A isoforms (E12 and E47) through alternative splicing, which differ only in the bHLH domain [4, 5]. Studies of different cells or diseases found that certain proteins are highly homologous or identical to E47 and E12, and they have been given new names, such as immunoglobulin transcription factor 1 (ITF-1) and vitamin D receptor interacting repressor (VDIR) [6, 7]. E2A consists of several activation domains (ADs) and a bHLH domain. The N-terminal ADs, which include ADs 1, 2 and 3 (AD1, AD2, and AD3, respectively), and the downstream eight-twenty-one (ETO)-interacting sequence (DES) domain are responsible for the transactivation functions of E2A. The bHLH domain is located in the C-terminus of E2A and is sufficient for dimerization and DNA binding activity [2, 8].

As a member of the E protein family, E2A also plays an important role in the immune system. In particular, E2A is required for normal T and B cells development. E2A generally functions as a transcriptional regulator and participates in the transcriptional network that affects lymphocyte development. Specifically, when E2A forms homodimers, it recruits a coactivator as a transcriptional activator, thereby causing transcriptional activation. However, when E2A forms heterodimers with class II bHLH proteins, it serves as a transcriptional activator or repressor that recruits the coactivator or corepressor, leading to transcriptional activation or suppression, respectively [2, 8, 9]. Over time, the major domains of E2A involved in transcriptional regulation have been gradually discovered, and the crystal or nuclear magnetic resonance (NMR) structures of these domains or key parts of these domains have been reported intermittently. However, the relationship between the domains of E2A and its role as a transcriptional regulator have rarely been summarized. In this review, the structural characteristics of each domain are described, and the common and respective characteristics of ADs and their transcriptional coactivator CREB-binding protein (CBP)/P300 and transcriptional corepressor ETO as transcriptional regulators of E2A, as well as the dimerization and E-box binding characteristics of the bHLH domain, are illustrated. This information will improve our understanding of the relationship between each domain and its function.

## The structure and function of AD1

### The structure of AD1

**Fig. 1:**
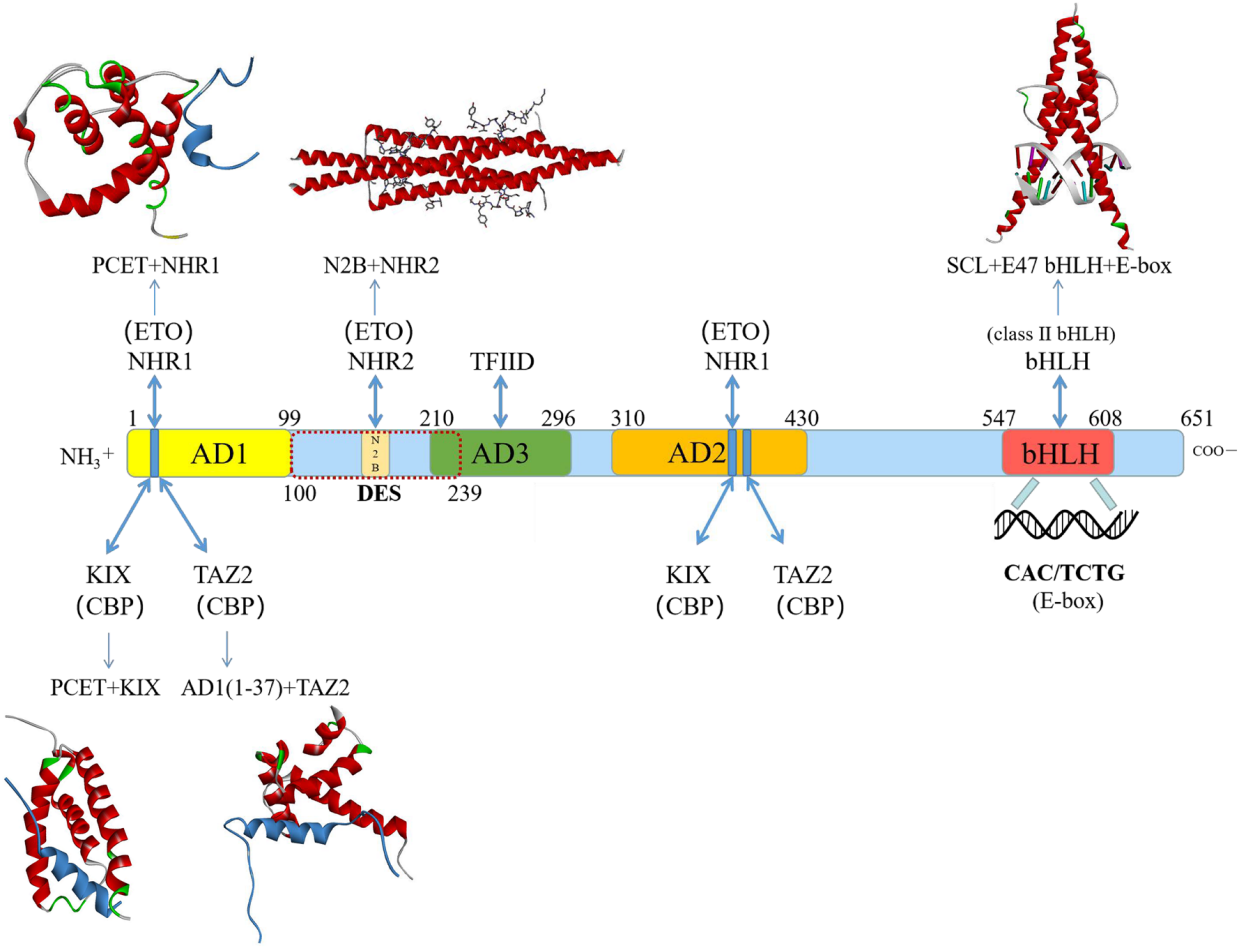
Architecture of the domains of E2A. The domain architecture of CBP/P300 is shown in the middle, illustrating the four activation domains (AD1, DES, AD3, and AD2) and the C-terminal bHLH domain. The yellow shaded box represents AD1, and the core PCET motif of AD1 is represented by the small blue rectangle in the yellow shaded box. The dotted box represents the DES domain, and the N2B motif is represented by the small gold rectangle in the middle of the dotted box. The green box represents AD3, the orange box represents AD2, and the red box represents the bHLH domain. The numbers indicate the positions of the residues at both ends of these domains, AD1 (residues 1–99), DES domain (residues 100–239), AD3 (residues 211–298), AD2 (residues 310–430) and bHLH domain (residues 547–608). The structures of each domain are also shown and labeled. Top panel: The E2A PCET motif in complex with the ETO NHR1 domain (PDB: 2KNH); the E2A N2B motif in complex with the ETO NHR2 domain (PDB: 4JOL); the structure of the complex formed between the E2A bHLH domain and the SCL bHLH domain and E-box (PDB: 2YPB). Bottom panel: The PCET motif in complex with the CBP KIX domain (PDB: 2KWF); the structure of the complex formed between E2A AD1 (residues 1–37) and the CBP TAZ2 domain (PDB: 2MH0). The structures of the complex formed between E2A AD2 and NHR1, NHR2 or TAZ2 and the complex composed of E2A AD3 and TFIID was unclear

E2A is expressed as two isoforms, E12 and E47, which are composed of approximately 600 amino acids. E2A contains five domains that are quite conserved in other E proteins (HEB and E2-2): ADs 1–3, the DES domain, and the bHLH domain. AD1 (amino acids 1–99) is located at the N-terminus of E2A. The DES domain (residues 100–239) is adjacent to AD1. AD3 (amino acids 211–298) and AD2 (amino acids 310–430) are in the middle of E2A. The bHLH domain (residues 547–608) is located at the C-terminus of E2A (Fig. 1) [10–13]. As transcriptional regulators, AD1, AD2, and AD3 of E2A and the NHR2-binding (N2B) motif mainly recruit transcriptional coregulators to initiate transcription, while the bHLH domain is mainly involved in the dimerization of E2A and binding to specific DNA motifs of target genes, leading to the execution of transcriptional regulation.

**Fig. 2:**
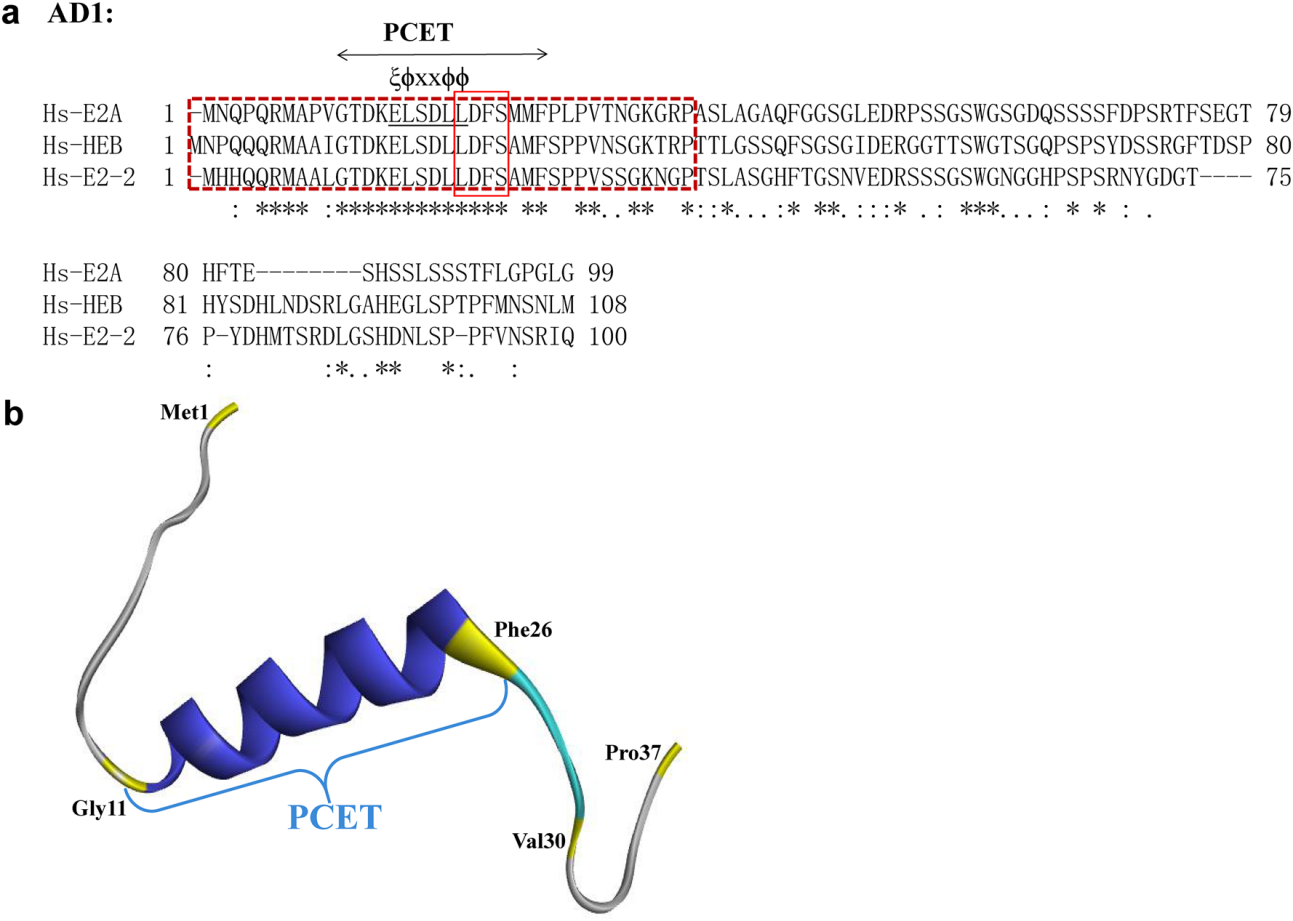
The structure of AD1. **a** Alignment of AD1 in human E proteins (E2A, HEB, and E2-2). The blue shaded box denotes the core PCET motif, and the bottom symbols represent “*” identical residues, “:” conserved residues, and “.” semiconserved residues. The red dashed box represents the part of AD1 where the 3D structure has been reported. The LDFS motif is indicated by the red box. The amino acid sequences of human E proteins (E2A, HEB, and E2-2) were propagated from UniProtKB (P15923-2, Q99081-1, and P15884-1). **b** The 3D structure of a portion of E2A AD1 (residues 1–37). The 3D structure was derived from the Protein Data Bank (PDB: 2MH0); the blue helix denotes the PCET motif and is labeled; the two coils are shown in gray, and the short extended region is in blue

Among these ADs of E2A, AD1 is the most frequently studied and was the first AD discovered. In 1993, Aronheim et al. first identified AD1 in E2A when studying the mechanism underlying the transcriptional activation of target genes caused by E2A [11]. AD1 of human E2A is composed of 99 amino acids at the N-terminus. As one of the most well-conserved domains among the E proteins, E2A AD1 consists of 38 identical residues, 28 conserved residues and 33 relatively variable residues when compared with other E proteins (HEB and E2-2) (Fig. 2a). The three-dimensional (3D) structure of the full-length E2A AD1 has not been solved, and only NMR structures of the 37 N-terminal residues of AD1 (Met1–Pro37) have been reported [14]. The N-terminal region of AD1 forms a structure in which an α-helix and an adjacent short extended region are sandwiched by two coils. The first coil consists of 10 N-terminal residues (Met1–Val10). The 16 residues (Gly11–Phe26) adjacent to the first coil form an amphipathic α-helix. Pro27–Pro29 form a short extended region. The remaining eight amino acids (Val30–Pro37) comprise the second coil. The α-helix makes an important contribution to binding to the coactivator CBP or the corepressor ETO protein; therefore, this α-helix is called the P300/CBP and ETO target in the E protein (PCET) motif [15]. The stable structure of the α-helix and its adjacent short extended regions are important contributors to the interaction between proteins and AD1, and these regions are also conserved in other E proteins (Fig. 2b).

The PCET motif is the core element of AD1 and the most highly conserved region among the AD1 of E proteins [14, 16, 17]. As a transcriptional regulator, the transactivation function of AD1 is generally achieved by binding to transcriptional coactivators or corepressors, such as CBP or ETO [18–20]. The PCET motif (Gly11-Phe26) includes 7 hydrophobic residues (Leu16, Leu19, Leu20, Phe22, Met24, Met25, and Phe26), 4 amino acids with intermediate polarity (Gly11, Thr12, Ser17, and Ser23), 4 acidic residues (Asp13, Glu15, Asp18, and Asp21) and a basic residue (Lys14). The contribution of each residue of PCET to the interaction between PCET and CBP or ETO is slightly different. The distributed hydrophobic residues (Leu16, Leu19, Leu20, Phe22, Met24, and Met25) and the nearby hydrophilic residues constitute the core elements of PCET and are called the nine amino acid transactivation domain (9aaTAD) motif [21, 22]. The 9aaTAD plays a major role in the interaction between PCET and CBP or ETO and consists of two overlapping protein recognition motifs, Glu–Leu–Ser–Asp–Leu–Leu (ELSDLL) and Leu–Asp–Phe–Ser (LDFS). ELSDLL consists of three leucines (Leu16, Leu19, and Leu20) and the polar residues near these leucines. The ELSDLL sequence, which belongs to the general ɸ-X-X-ɸ-ɸ sequence, is present in the ADs of many transcription factors [18, 23, 24], where ɸ represents a bulky hydrophobic residue and X represents any amino acid residue. The four consecutive residues (residues 20–23) in the central portion of the 9aaTAD constitute the LDFS motif (Fig. 2a) [16, 25].

### Interactions with CBP and ETO

**Fig. 3:**
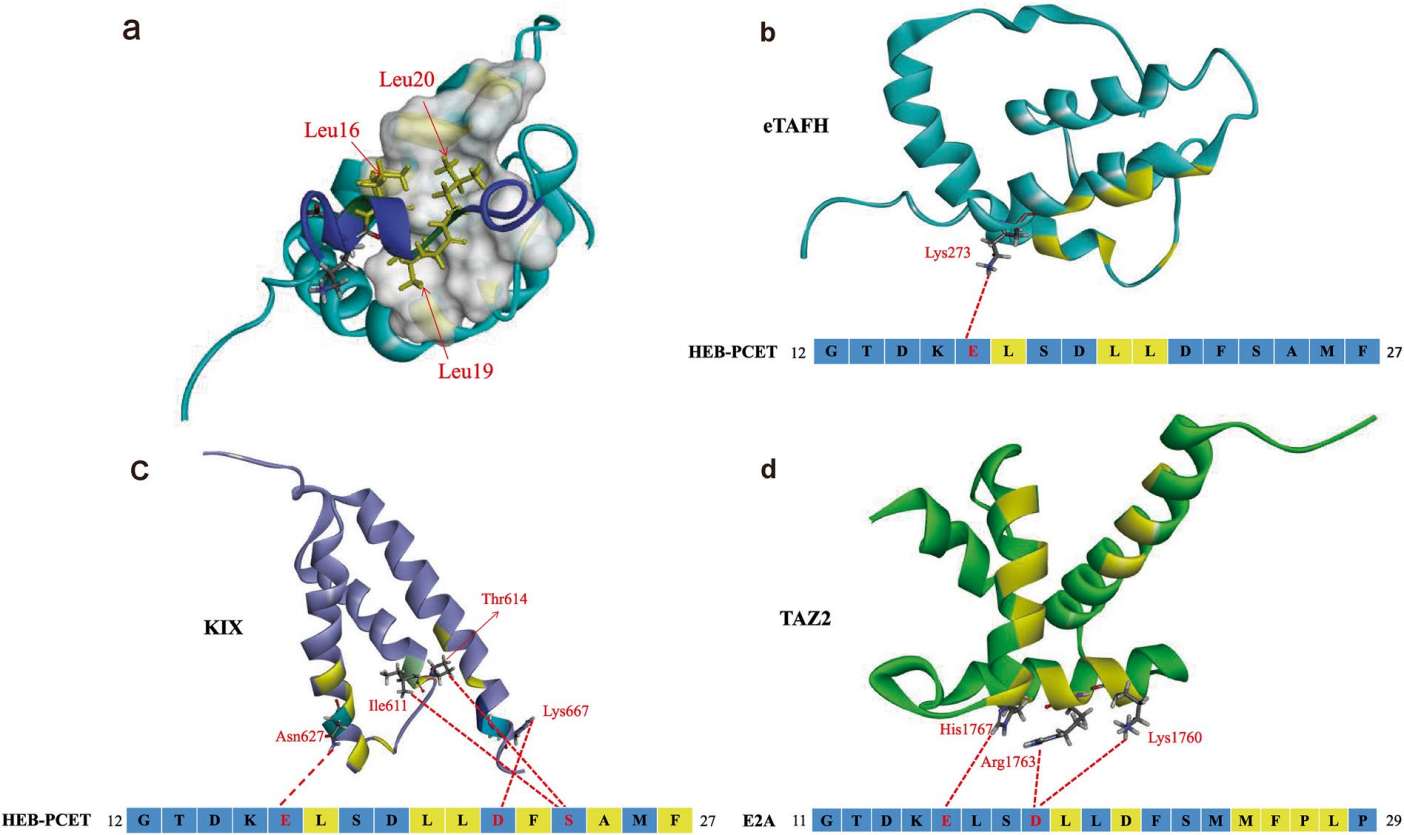
The interaction of AD1 with CBP or ETO. **a** Sharing mode of the interaction of AD1 with the domains of CBP or ETO. The residues Leu16, Leu19, and Leu20 of E2A are shown and labeled; the hydrophobic grooves on the surface of the domains of CBP or ETO are shown in gray and white. The 3D structure was obtained from the Protein Data Bank (PDB: 2MH0) and was modified using Discovery Studio 3.0 software. **b** Characteristics of the interaction of AD1 with NHR1/eTAFH. Top panel: The structure of eTAFH was obtained from the Protein Data Bank (PDB: 2KNH); the region marked in yellow on the domains represents the hydrophobic residues in the domains that bind to AD1; the polar residues on the domains that bind to AD1 are shown and labeled in red. Bottom panel: The sequences of HEB-PCET (12–27) were obtained from the Protein Data Bank (PDB: 2KNH); the hydrophobic residues in the sequences that contribute to the interaction with the NHR1/eTAFH domain of ETO are represented by yellow squares, and the polar residues involved in the interaction are marked in red. The red dotted line represents hydrogen bonds. **c** Top panel: The structure of KIX was obtained from the Protein Data Bank (PDB: 2KWF); the region marked in yellow on the domains represents the hydrophobic residues in the domains that bind to AD1; the polar residues in the domains that bind to AD1 are shown and labeled in red. Bottom panel: The sequences of HEB-PCET (12–27) are shown; the hydrophobic residues in the sequences that contribute to the interaction with the KIX domain of CBP are represented by yellow squares, and the polar residues involved in the interaction are marked in red. The red dotted line represents hydrogen bonds. **d** Top panel: The structure of the TAZ2 domain was obtained from the Protein Data Bank (PDB: 2MH0); the region marked in yellow on the domains represents the hydrophobic residues in the domains that bind to AD1; the polar residues of the domains that bind to AD1 are shown and labeled in red. Bottom panel: The sequences of E2A-PCET (11–29) were obtained from the Protein Data Bank (PDB: 2MH0); the hydrophobic residues in the sequences that contribute to the interaction with the KIX domain of CBP are represented by yellow squares, and the polar residues involved in the interaction are marked in red. The red dotted line represents hydrogen bonds

As a transcriptional regulator, E2A recruits CBP or ETO to the promoter of acetylate or deacetylate histones, thereby leading to chromatin remodeling. The recruitment of CBP or ETO by E2A is mainly achieved through the interaction between the ADs of E2A and domains of CBP or ETO [26, 27]. AD1 generally interacts with the kinase-inducible domain interacting (KIX) domain and transcription adaptor putative zinc finger (TAZ2) domain of CBP and the TATA box-binding protein (TBP)-associated factor (TAF) homology (eTAFH, also called Nervy homology domain 1 (NHR1)) domain of ETO. The PCET motif is almost identical in E proteins. Based on the NMR structures of compounds containing AD1, namely, the E2A-AD1:TAZ2 complex, HEB-PCET:KIX complex, and HEB-PCET:eTAFH complex (Fig. 3) [14, 28, 29], the interactions between AD1 and these domains of CBP and ETO have many commonalities and some subtle differences.

The commonalities are that the hydrophobic residues of the core PCET motif of AD1 insert into the hydrophobic groove on the surface of KIX, TAZ2 or NHR1; the hydrophobic residues of E2A interact with the amino acids in the hydrophobic groove through many nonpolar forces; and a small number of polar residues on PCET generate hydrogen bonds and electronic forces between residues of these domains to complement those nonpolar forces. More specifically, Leu16, Leu19 and Leu20 of PCET interact separately with residues in the three small hydrophobic pockets that comprise the hydrophobic groove (Fig. 3a), and the polar residue Glu15 interacts with polar residues outside the hydrophobic groove, namely, Asn627 of KIX, K273 of NHR1, and His1767 of TAZ2, through hydrogen bonds [14, 28, 29].

In addition, the interaction of AD1 with KIX, TAZ2 or NHR1 has its own characteristics. When PCET interacts with NHR1, perhaps due to a defect in the structure detection method, only Glu15, Leu16, Leu19 and Leu20 interact with the residues along the hydrophobic groove of NHR1 (Fig. 3b) [28]. When PCET interacts with KIX, in addition to Glu15, Leu16, Leu19 and Leu20 described above, the hydrophobic residues Phe22, Met24 (Ala25 in HEB), and Phe26 of PCET also interact with the residues in the hydrophobic cleft between helices H1 (Ile611, Phe612, Ala619, and Arg624) and H3 (Leu628, Tyr631, Ile660, Leu664, and Lys667) of KIX. The polar residue Asp21 of PCET interacts with Lys667 of KIX by forming a salt bridge, and Ser23 of PCET interacts with Thr614 of KIX by forming hydrogen bonds. A salt bridge also forms between Lys14 and Asp18 of E2A. Ser17 of PCET is exposed to solvent and does not contact any KIX residue. Gly11–Asp13 of PCET may have little contact with KIX due to flexibility (Fig. 3c) [29]. However, compared with the interaction between HEB-PCET and KIX, Leu20, Ser23, and Met24 are not involved in the interaction between E2A-AD1:TAZ2, while Asp18 and Met25 of PCET and Pro27 and Leu28 near the PCET motif are involved (Fig. 3d) [14]. In addition, the key residues of AD1 that bind to KIX, NHR1 or TAZ2 are also different. Leu20 in E2A is essential for E2A:KIX and E2A:eTAFH interactions [28, 29]. Leu19 and Phe22 in E2A are essential for E2A:TAZ2 interactions [14]. Key and major residues contributing to these protein interactions are located in the LDFS and 9aaTAD motifs. In addition, the three residues of the short extended region (Phe26, Pro27 and Leu28) contact the TAZ2 domain through extensive nonpolar contacts. Due to the insufficient details of the NMR structure of the known PCET-KIX complex and PCET-NHR1 complex, the role of the short extended region in the E2A:KIX and E2A:eTAFH interactions is unclear.

Although AD1 interacts with multiple domains on CBP and ETO, the roles of KIX, NHR1, TAZ2 and AD1 are generally independent and cooperative. The NHR1 binding site on AD1 is consistent with the KIX binding site, and the KIX domain of CBP and the NHR1 domain of ETO competitively bind AD1. Although both the KIX and TAZ2 domains of CBP bind to AD1, the key residues in their identified regions that bind to AD1 are slightly different; Leu20 is the key residue for the KIX interaction, whereas Leu19 and Phe22 are the key residues for the TAZ2 interaction [14, 28–30].

## The structure and function of other ADs

**Fig. 4:**
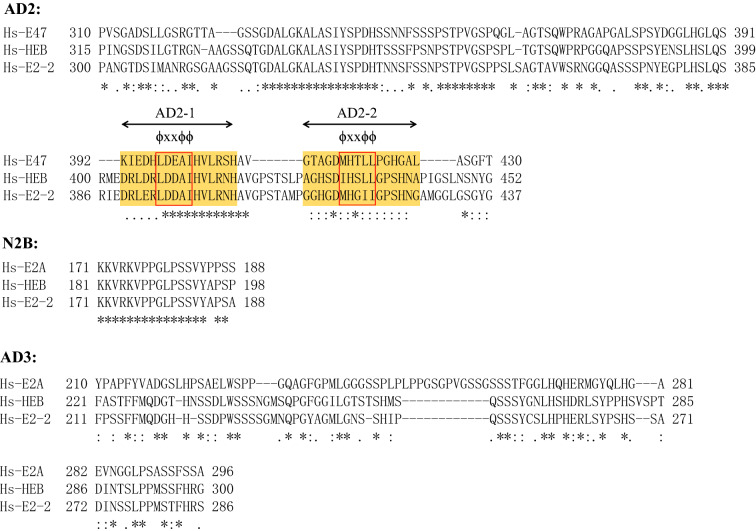
Alignment of other ADs of human E proteins (E2A, HEB, and E2-2). The orange shaded box denotes the core motif, and the bottom symbols represent “*” identical residues, “:” conserved residues, and “.” semiconserved residues. The ɸ-X-X-ɸ-ɸ motif is shown in the red box. The amino acid sequences of human E proteins (E2A, HEB, and E2-2) were propagated from UniProtKB (P15923-2, Q99081-1, and P15884-1)

In addition to AD1, E2A contains three other ADs, namely, AD2, AD3, and DES. AD2 is located in the central region of the E2A protein. In the detailed mechanism of E2A activation and transcription reported by Aronheim et al., AD2 was first identified together with AD1 [11]. AD2 is also conserved among the E proteins [11]. Compared with AD2 of HEB and E2-2, human E2A AD2 (residues 310–430) consists of 66 identical residues, 15 conserved amino acid residues and 40 relatively variable residues. AD2 contains two ɸ-X-X-ɸ-ɸ motifs that are similar to the PCET motif of AD1, AD2-1 (Lys392–His407) and AD2-2 (Gly410–Leu425) (Fig. 4a) [32]. AD2 of E2A interacts with CBP/P300 either independently of or cooperatively with AD1 to promote transcriptional activation [31–33]. In addition, AD2 also binds to the hydrophobic surface of the KIX domain of CBP/P300 and the binding region of AD1, mainly through the ɸ-X-X-ɸ-ɸ sequence (LDEAI) on AD2-1. In addition, AD1 and AD2 of E2A also bind to the TAZ2 and KIX domains of CBP/P300 or NHR1 of ETO in a cooperative manner [14, 28, 29].

The DES domain (residues 100–239) is adjacent to AD1. It is another conserved ETO-interacting region that was discovered by Guo et al. in 2009 [12]. NHR2 binds to an identical sequence in the DES domain of the E proteins. Previous studies have suggested that ETO binds to AD1 of the E proteins through the NHR1 domain, and Sun et al. found that NHR2 of acute myeloid leukemia 1 protein (AML1)-ETO also plays a role in leukemogenesis and identified an N2B motif for the first time [34]. This motif contains 18 residues and is basically the same in other members of the E protein family (E2A, K171-S188; HEB, K181-S198; and E2-2, K171-S188) (Fig. 4). The crystal structure of the N2B-NHR2 complex contains N2B and NHR2, and site-directed mutagenesis of residues in N2B showed that Pro187, Pro191 and Ser192 play key roles in the interaction between N2B and NHR2 [34–36].

AD3 is located between the DES domain and AD2. Chen et al. studied the mechanism by which transcription factor II D (TFIID) functions as a coactivator through direct interactions with E proteins. TFIID is a multi-subunit complex composed of TBP and TAFs and was first discovered in AD3 of the E proteins [13]. E proteins enhance the recruitment of TFIID and the binding of the TATA box to the promoter through AD3 binding to the TAFH4 domain of TAF3 (a component of TFIID), thereby promoting the transcription of the target gene. Among the members of the E protein family, AD3 of HEB and E2-2 are highly conserved, but the homology of E2A and HEB AD3 (221–300) is only approximately 30% (Fig. 4). Although their homology is different, they all play roles in the recruitment of TFIID through AD3 [13, 37, 38]. Therefore, we hypothesize that several highly conserved protein recognition motifs may be present in AD3 of the E proteins, which may be detected in the future.

## The structure and function of the bHLH domain

### The structure of the bHLH domain

**Fig. 5:**
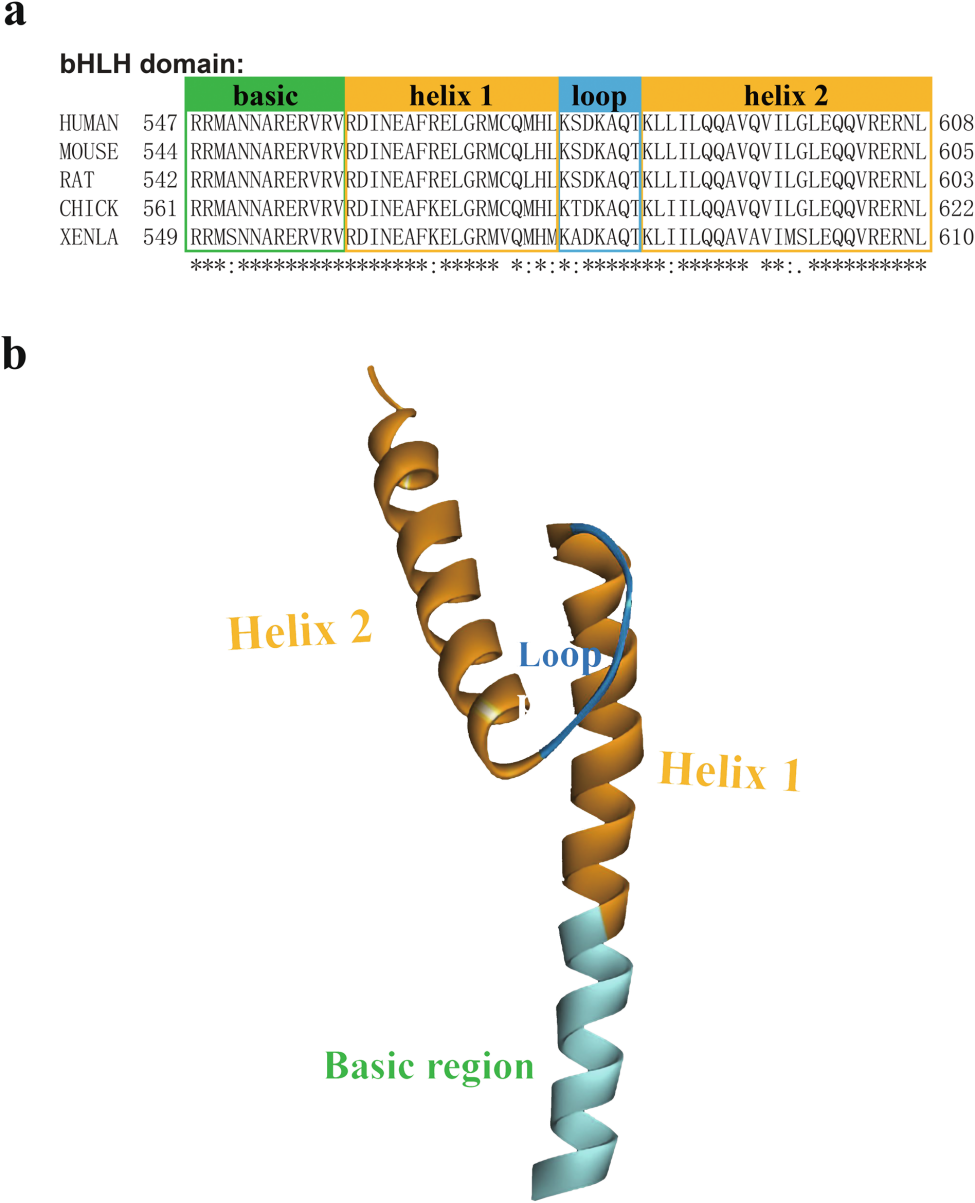
Structure of the bHLH domain. **a** Sequence alignment of the bHLH domain of E47 from humans, rats, mice, Xenopus and chickens. Shaded boxes denote the amino acid sequence of each motif, “basic region, helix 1, loop, and helix 2”, and the bottom symbols represent “*” identical residues, “:” conserved residues, and “.” semiconserved residues. The amino acid sequences of the bHLH domain of E47 from humans, rats, mice, Xenopus and chickens were propagated from UniProtKB (P15923-2, P15806-2, P21677-2, Q70IK2-1, and Q01978-2, respectively). **b** The 3D structure of the bHLH domain of human E47. The area of each motif in the bHLH domain is displayed with the “basic region, helix 1, loop and helix 2”. The “basic region” is shown in blue, “helix 1” and “helix 2” are both shown in orange, and the “loop” is shown in dark blue. This 3D structure was obtained from the Protein Data Bank (PDB: 2YPB)

E2A is a member of the bHLH transcription factor family that shares the bHLH domain and thus plays a vital role in regulating cell proliferation, differentiation and development, especially lymphoid development. The two E2A isoforms, E12 and E47, differ only in the bHLH domain [2, 8]. Some studies have shown that the affinity of E12 for DNA is significantly lower than that of E47 [39]. The reported crystal structure of the bHLH domain of E2A is basically based on the bHLH sequence of E47. The crystal structure of the E2A bHLH domain was first determined by Ellenberger et al. in 1994 [40]. The bHLH domain contains an adjacent N-terminal basic region (b) that contacts the major groove of the DNA and binds to a typical DNA motif termed the E-box (CANNTG) and a C-terminal HLH region that participates in interacting with the E47 dimer [2, 8]. As an essential part of E2A, the bHLH domain is sufficient for dimerization and DNA binding activities and is highly conserved among species. The sequence alignment of the E47 bHLH domain between human, mouse, rat, chicken and *Xenopus* proteins showed that this domain consisted of 52 identical amino acid residues and 10 relatively conserved residues. The basic region is almost identical to the E47 bHLH domain of humans, rats, mice, chickens and *Xenopus*, except for Ser552 in the Xenopus protein, which is replaced with Ala (A) in proteins from other species. Most residues of the HLH region in the VDIR bHLH domain are identical among these species, and a few conserved or variable residues are scattered in several motifs (helix 1, helix 2, and loop) (Fig. 5a).

The human E47 bHLH domain (residues 547–608) consists of two helixes and a loop located in the middle of these two helixes. The first helix is composed of 31 amino acid residues from the N-terminus of the bHLH domain. This helix includes a basic region and helix 1 of the HLH region. The basic region is composed of 13 amino acid residues from the N-terminus of the first helix (Arg547–Val559). Helix 1 is formed from 18 residues near the C-terminus of the first helix (Arg560–Leu577). The loop structure consists of seven amino acid residues adjacent to helix 1 (Lys578–Thr584). The remaining residues in the bHLH domain (Lys585–Leu608) constitute the other helix (called helix 2) (Fig. 5) [40, 41].

## Structural insights into the function of the bHLH domain

**Fig. 6:**
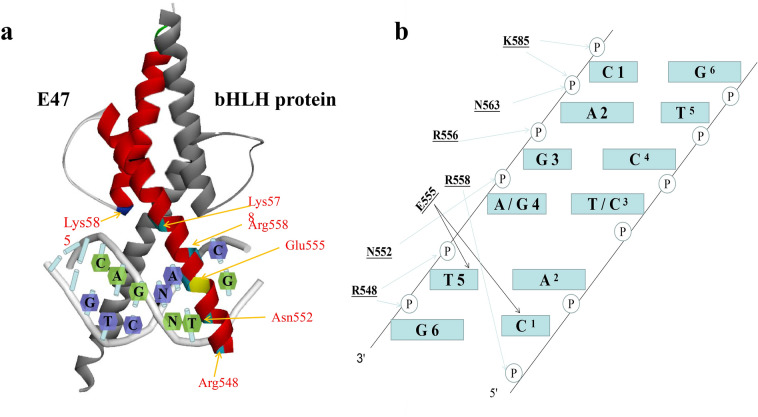
The interaction of a bHLH domain with an E-box. **a** 3D structure of the complex of the E47 dimer and the E-box. The spatial location of residues that participate in binding to the E-box (Arg548, Asn552, Glu555, Arg556, Arg558, Asn563 and Lys585) is shown; these amino acids are marked in blue or yellow and are shown as abbreviations. “bHLH protein” mainly indicates E proteins or class II bHLH proteins. The 3D structure was obtained from the Protein Data Bank (PDB: 2YPB) and was modified using Discovery Studio 3.0 software. **b** Pattern chart of the interaction of the residues on the bHLH domain with the E-box. The DNA phosphate backbones are represented as filled circles, and the bases are represented as “C, A, T, and G,” such as “C 1,” which represents the cytosine at position 1 on a strand of the E-box. The residues on the bHLH domain are Arg548, Asn552, Arg558, Asn563, and Lys585. The light arrows represent the interactions between the residues and the DNA backbones, and the black arrows represent the interactions between the residues and the bases

The bHLH domain of E2A usually initiates transcription by forming dimers with homologous proteins or other bHLH proteins and then binding to the E-box on the promoter of target genes. When E2A forms dimers with different proteins, the dimers bind to diverse E-boxes. The E47 homodimer preferentially binds to the E-box sequence CACCTG, whereas the E47-class II bHLH protein preferentially binds to the E-box sequence CATCTG [42, 43]. In general, the bHLH domain of the E47 monomer binds independently to its preferred DNA base. The crystal structure of the E47 bHLH-DNA complex has been determined; the HLH regions of the E47 dimer form a four-helix bundle with basic regions inserted into the DNA, and the pseudodyad of the DNA is almost parallel to the twofold axis of the basic region (Fig. 6a). This conformation is similar to other HLH proteins/DNA complexes, such as myoblast determination protein (MyoD)/DNA (PDB ID: 1MDY) [44, 45].

Several researchers have solved the crystal structure of E47–E47/DNA complexes and E2A heterodimer-DNA complexes [40, 41, 46, 47]. Based on these crystal structures, the DNA binding characteristics of E2A have been identified. Most residues in the E2A bHLH participate in dimerization or in the recognition of and binding to DNA. The residues that participate in the recognition of and binding to the E-box are Arg548, Asn551, Asn552, Glu555, Arg556, Arg558, Asn563 and Lys585. Asn563 (helix 1) and Lys585 (helix 2) are located in the HLH region, while the other residues are located in the basic region. Arg556 seems to guide the E2A bHLH to bind to a specific E-box (Fig. 6a). When the basic region helix is inserted into the DNA groove, Arg556 generally interacts with the two central phosphate backbones on the opposite strand of the preferred E-box, CAGGTG or CAGATG. At this position, glutamate is highly conserved in many bHLH proteins and thus makes important contributions to the binding of the protein to a specific E-box. Glu555 of E2A normally interacts with the cytosine and adenine of the preferred half of the E-box (CAC/CAT) [2, 40, 41, 47]. The scattered interaction with the phosphate backbone is the main force stabilizing the bHLH-DNA interaction, and the residues involved in these interactions include Arg548, Asn552, Arg558, Asn563, and Lys585. Arg548 contacts the phosphate groups of two 3ʹ flanking nucleotides (TG) on E-box-b, which is the opposite strand of the preferred E-box. Asn552 contacts the backbone at position 4 or 5 in E-box-b. Arg558 binds to the phosphate backbone of the first site (C) of E-box-a, the preferred E-box. Arg558 stabilizes the position of Glu555 by forming a salt bridge that exists in many bHLH domains. Asn563 in helix 1 contacts the phosphate groups at position 2 (A) in E-box-b. Lys585 in helix 2 contacts the phosphate backbone of (C) and (A), which is located on the 5ʹ flank of E-box-b. Moreover, Arg554 interacts with nucleotides near the E-box and contributes to the stabilization of the bHLH-DNA complex. In the E2A homodimer-DNA interaction, N551 on a monomer also interacts with the base (G) at the end of the E-box (Fig. 6b) [2, 40, 41, 47].

### Interaction with the bHLH dimer

**Fig. 7:**
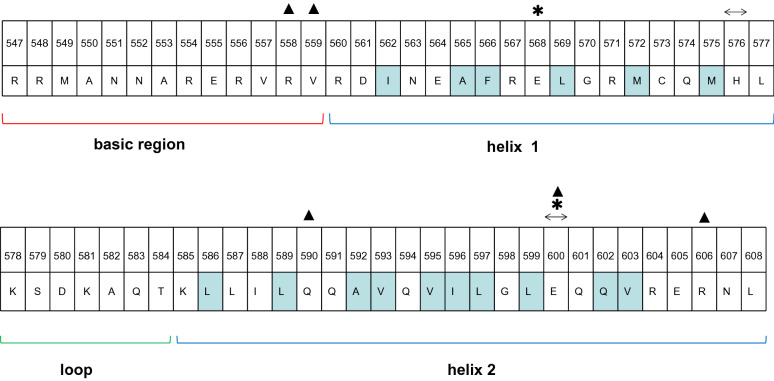
The interaction within the E2A bHLH dimer. Shaded residues are in the dimer interface: hydrophobic residues are in blue. The residues involved in hydrogen bonding are marked with symbols: ↔, E2A homodimer; *, E2A-class II bHLH heterodimers; ▲, E2A–ID1 heterodimer. Amino acid positions 547–608 of E47 were obtained from UniProtKB (P15923-2)

The HLH region of the bHLH domain is involved in the homo- or heterodimerization of E2A. The dimerization of E2A is generally stabilized by hydrophobic forces, hydrogen bonds and van der Waals interactions between the HLH regions in the two monomers. The specific forces contributing to this stable dimerization between homodimers and heterodimers are slightly different (Fig. 7). First, the hydrogen bonds between the bHLH domains of the E2A homodimer are symmetrically positioned. Glu600 of helix 2 from one monomer contacts His576 of helix 1 from the other monomer through hydrogen bonds. When forming a heterodimer with class II bHLH proteins, Glu600 (helix 2, H2) of E2A interacts with tyrosine in the first position of helix 2 of the class II bHLH protein through hydrogen bonds. Since the tyrosine at this position is a conserved residue in class II bHLH proteins, this hydrogen bond may be a common feature of E2A-class II bHLH heterodimers [2, 47]. In addition, Glu568 of E2A interacts with Lys (position 2 of helix 2, H2.2) of a few class II bHLH proteins (such as NeuroD1) through hydrogen bonds. Hydrophobic forces are generally the main dimer-forming interactions. The residues of E2A involved in hydrophobic interactions include Ile562, Ala565, Phe566, Leu569, Met572, Met575, Leu586, Leu589, Ala592, Val593, Val595, Ile596, Leu597, Leu599, Gln602 and Val603. The packing interactions are second only to the hydrophobic interactions in terms of stabilizing the dimer structure. The residues of E2A that participate in the packing interactions of homologous dimers are Asp561, Ile563, Ala565, Phe566, Glu568, Leu569, Met572, His576, Lys585, Leu586, Leu589, Gln590, Ala592, Val593, Val595, Ile596, Leu597, Leu599, Glu600, Gln602 and Val603. The residues of E2A involved in the packing interactions of heterogeneous dimers are mostly the same as those of homologous dimers. In addition, some bHLH II proteins (such as NeuroD1) lose filling interactions due to a shorter helix 1. Some bHLH II proteins (such as NeuroD1) contain smaller residues (Leu7 of helix 1, H1.7, and Ser16 of helix 2, H2.16) that pack less tightly than the larger residues of E2A (Phe7 of helix 1, H1.7, and Glu16 of helix 2, H2.16). However, the monomeric structure of the E2A bHLH domain is mainly stabilized by the hydrogen bond network between helix 1, helix 2 and the loop of the HLH region. This hydrogen bond network is composed of hydrogen bonds between residues of two monomers, such as Asn563–Lys585, Gly570–Gln583, Gln574–Lys581, Gln583–Gln591, and Thr584–Leu587 [40, 41, 47].

In addition to class II bHLH proteins, E2A proteins can dimerize with inhibitor of DNA binding (ID) proteins. ID proteins are also known as the class V helix-loop-helix (HLH) family. Unlike class II bHLH proteins, the HLH domain of ID proteins lacks a key DNA recognition region. When E2A dimerizes with ID proteins, it cannot bind to DNA stably, resulting in the failure of E2A transcriptional regulation [8, 48]. Different from class II bHLH proteins, there are six hydrogen bonds and two salt bridges in the interface area of the E2A–ID1 dimer. The six hydrogen bonds are ID1-L59: E47-Q590, ID1-Q89: E47-R558, ID1-Q89: E47-V559, ID1-Y94: E47-E600, ID1-L102: E47-R606, and ID1-S104: E47-R606. E600 is also involved in the formation of hydrogen bonds in the E47 homodimer and heterodimer. The two salt bridges are ID1-R99: E47-E568 and E47-E568: E47-R571 [48]. Moreover, structural changes in E2A can affect the development of diseases. For example, the genes encoding the transcription factor TCF3 (E2A) and its negative regulator ID3 belong to the most frequently mutated genes in Burkitt lymphoma. Four evolutionarily conserved amino acids were the target of most of the E47 missense mutations, which occurred in both the basic DNA recognition region (N551K, V557E/G) and in the HLH region (D561E/V/N, M572K). E47 missense mutations could disrupt intra- or intermolecular interactions [49].

## Conclusions

Overall, E2A functions as a transcription regulator, which requires the collaborative participation of ADs and the bHLH domain. First, E2A forms a dimer with bHLH proteins through their HLH regions, and the hydrophobic residues on the HLH region play important roles in the formation of the dimer, followed by AD1 binding to the KIX or TAZ2 domain of CBP through the PCET motif. Thereafter, CBP presents histone acetyl-transferase activity, and the promoter region is exposed [14, 28, 29, 50]. The major process underlying the interaction of PCET-KIX is that the three leucine residues present in the LXXLL sequence of PCET pack into the hydrophobic pocket of KIX. Finally, the basic helix of the E2A bHLH domain binds to the exposed E-box and initiates transcription, and a key residue of the bHLH domain, Glu555, normally interacts with the cytosine and adenine of the preferred half of the E-box (CAC/CAT) [2]. Moreover, AD3 of E2A binds to the TAFH4 domain of the basal transcription factor TAF3 and thus causes the transcription of target genes [13]. However, in the presence of a high level of ETO, ETO competes with CBP for binding to AD1 of E2A through NHR2. Next, histone deacetylases are recruited, and transcriptional repression occurs [12, 50, 51]. Although the 3D structure of part of the E2A domain has been reported, the full-length 3D structure of the four ADs of E2A has not yet been elucidated. Future discoveries of the full-length structures of ADs will help determine the function of E2A. Protein–protein interactions (PPIs) are ubiquitous in biological systems and are often misregulated in diseases. Sawyer et al. used secondary and tertiary protein domain mimics (PDMs) to specifically modulate PPIs and then developed new PPI-based therapeutics [52]. E2A plays a key role in the pathogenesis of leukemia. It will be a new strategy for the treatment of leukemia to generate PDMs that target the PPIs associated with the key motif of E2A with high affinity and specificity. Moreover, based on the analysis of the interaction between E47 and ID1 protein, Wojnarowicz and others found the antagonist of the ID protein family, AGX51 [48]. This molecule was identified in an in silico screen for compounds that could interact with a hydrophobic pocket within the highly conserved loop. The region of the ID HLH dimerization motif has been verified to be the perturbation of the ID1/E47 interaction. In the future, the corresponding small molecule inhibition can be developed through the hydrophobic pocket within the E47 dimerization motif or the hydrophobic pocket of E47 partner class II bHLH proteins, potentially providing new therapeutic drugs for the treatment of leukemia involving E47.

## Data Availability

Not applicable.

## Abbreviations

ITF-1: Immunoglobulin transcription factor 1
VDIR: Vitamin D receptor interacting repressor
ADs: Activation domains
DES: Downstream ETO-interacting sequence
bHLH: Basic helix-loop-helix
CBP: CREB-binding protein
ETO: Eight twenty-one
PCET: P300/CBP and ETO target in E proteins
9aaTAD: 9 Amino acid transactivation domain
KIX: Kinase-inducible domain interacting
eTAFH: ETO TATA box-binding protein associated factor homology
NHR1: Nervy homology region 1
TAZ2: Transcriptional adapter zinc finger 2
NHR2: Nervy homology region 2
N2B: NHR2-binding
TFIID: Transcription factor IID
TBP: TATA box binding protein
HLH: Helix-loop-helix
TAFH4: TATA-binding protein associated factor 4
PPIs: Protein–protein interactions
PDMs: Protein domain mimics
